# Development of a Nanoscale Protein–Protein Mapping of PDE4 Interface-Disrupting Peptides

**DOI:** 10.1021/acs.nanolett.6c01869

**Published:** 2026-07-01

**Authors:** Sibo Lyu, Aleksandra Judina, Jiayue Ling, Thomas A Wright, Aditi Kulkarni, Jiarong Fu, Iyobel Kibreab, George Baillie, Prashant Kumar Srivastava, Julia Gorelik

**Affiliations:** † Cardiac Section, National Heart and Lung Institute (NHLI), Faculty of Medicine, 156647 Imperial College London, Hammersmith Campus, Du Cane Road, London W12 0NN, U.K.; ‡ School of Cardiovascular and Metabolic Health, 3526 University of Glasgow, University Avenue, Glasgow G12 8QQ U.K.; § School of Cancer Sciences, University of Glasgow, University Avenue, Glasgow G12 8QQ, U.K.

**Keywords:** Disruptor peptide, protein−protein interaction, computational framework, PDE

## Abstract

Spatially confined β-adrenergic receptor-cAMP nanodomain signaling depends on scaffolded protein–protein interactions (PPIs), yet converting such nanointerfaces into cell-active disruptor peptides remains challenging. Here, we identify a previously unrecognized phosphodiesterase 4A (PDE4A)-filamin A complex in human cardiac tissue that is disrupted in dilated cardiomyopathy. To target this interaction, we developed a nanodomain-resolved AlphaFold3 workflow integrating interface-recurrence filtering, orthogonal docking, and peptide-binding site inference to define a tractable binding region. This approach identified a filamin A docking sequence spanning R2520-H2528, which was optimized to RLVSNHSLH and rendered cell-permeant by N-terminal polyarginine tagging. In ventricular cardiomyocytes, the peptide reduced PDE4A–filamin A proximity and selectively attenuated β-adrenergic cAMP signaling in cytosolic and sarcolemmal compartments, measured by FRET biosensors. This work establishes a potential transferable strategy for translating predicted scaffolded PPI nanointerfaces into functional disruptor peptides and highlights compartmentalized signaling complexes as actionable targets for selective cellular modulation.

## Graphical abstract

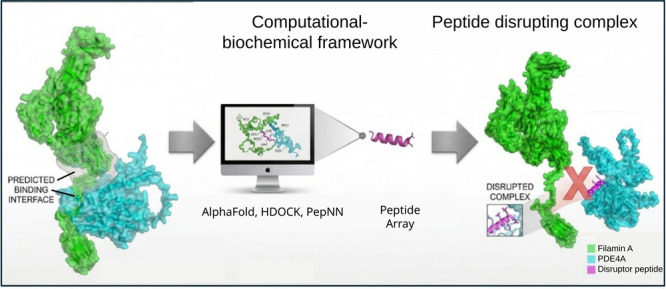

Chronic heart failure affects over 64 million people globally, with dilated cardiomyopathy (DCM), characterized by left ventricular dilation and impaired contraction, accounting for nearly one-third of all cases.^1–3^ Cardiac contraction depends on precisely coordinated neurohumoral activation via β-adrenergic receptors (βARs) and protein–protein interaction (PPI) networks that regulate excitation-contraction coupling, signal transduction, and structural integrity within cardiomyocytes.^4^

Among the critical regulators of βAR signaling are cAMP-specific phosphodiesterases (PDEs), which maintain localized control of intracellular second messenger cAMP gradients.^5^ PDE4A plays a pivotal role in shaping compartmentalized cAMP response in healthy cardiomyocytes.^6,7^ Decreased PDE4A activity, reported in patients with cardiac hypertrophy and DCM, results in hyperphosphorylation of protein kinase A (PKA) substrates, abnormal calcium cycling, and impaired cardiac function.^7,8^

Recent studies have highlighted the importance of anchoring PDE4A, among other PDE4s, to specific subcellular domains via interactions with scaffold proteins.^9,10^ Filamin A interacts with PKA, adenylate cyclase (AC), positioning it at the intersection of βAR signaling and mechanical stress responses in cardiomyocytes.^11–13^ Filamin A is also subject to a wide range of post-translational modifications; however, the role of PDE4s, key regulators of cardiomyocyte cAMP activity, within the filamin A complex has not been established.^14,15^ Disruption of other PDE4-protein complexes, such as PDE4-heat shock protein 20 (HSP20) and PDE4-Popeye domain-containing proteins have been implicated in cardiac hypertrophy and cardiac pacemaking, suggesting that selective targeting of PDE4 scaffolding interactions may modulate disease phenotype.^9,10^

Here, we utilized a biochemical approach to identify a novel interaction between filamin A and PDE4A. Next, to investigate the functional significance of this interaction, we developed a new computational pipeline to generate a disruptor peptide that selectively interferes with the PDE4A–filamin A binding interface.

Traditional biochemical methods for mapping PPIs are often costly, lengthy, and low-throughput, whereas bioinformatics-guided peptide design provides an efficient approach to identifying and targeting specific molecular interfaces. In this study, we combined in silico predictions with experimental validation to develop a disruptor peptide that selectively interferes with the PDE4A–filamin A interaction.

Using structural and motif-based analyses, we identified the minimal filamin A domain responsible for PDE4A binding and employed molecular modeling and peptide docking to screen candidate peptides that could compete with the PDE4A–filamin A interface. The peptides were predicted to avoid the annotated catalytic region, consistent with an interface-directed design. PDE4A enzymatic activity was not directly measured.

The most promising candidate was validated through biochemical assays and physiological experiments in cardiomyocytes. The disruptor peptide, delivered via a cell-permeable tag, enabled targeted disruption of the PDE4A–filamin A interaction and enabled evaluation of its contribution to cardiac signaling and function. We propose that the method described here can provide a new tool for studying maladapted signaling that underpins the modulation of cardiac function in disease.

To study the potential role of the PDE4A–filamin A complex in maladaptive cardiomyocyte remodeling, we designed a study pipeline incorporating biochemical, bioinformatic and physiological experiments (Figure 1).

**Figure 1:**
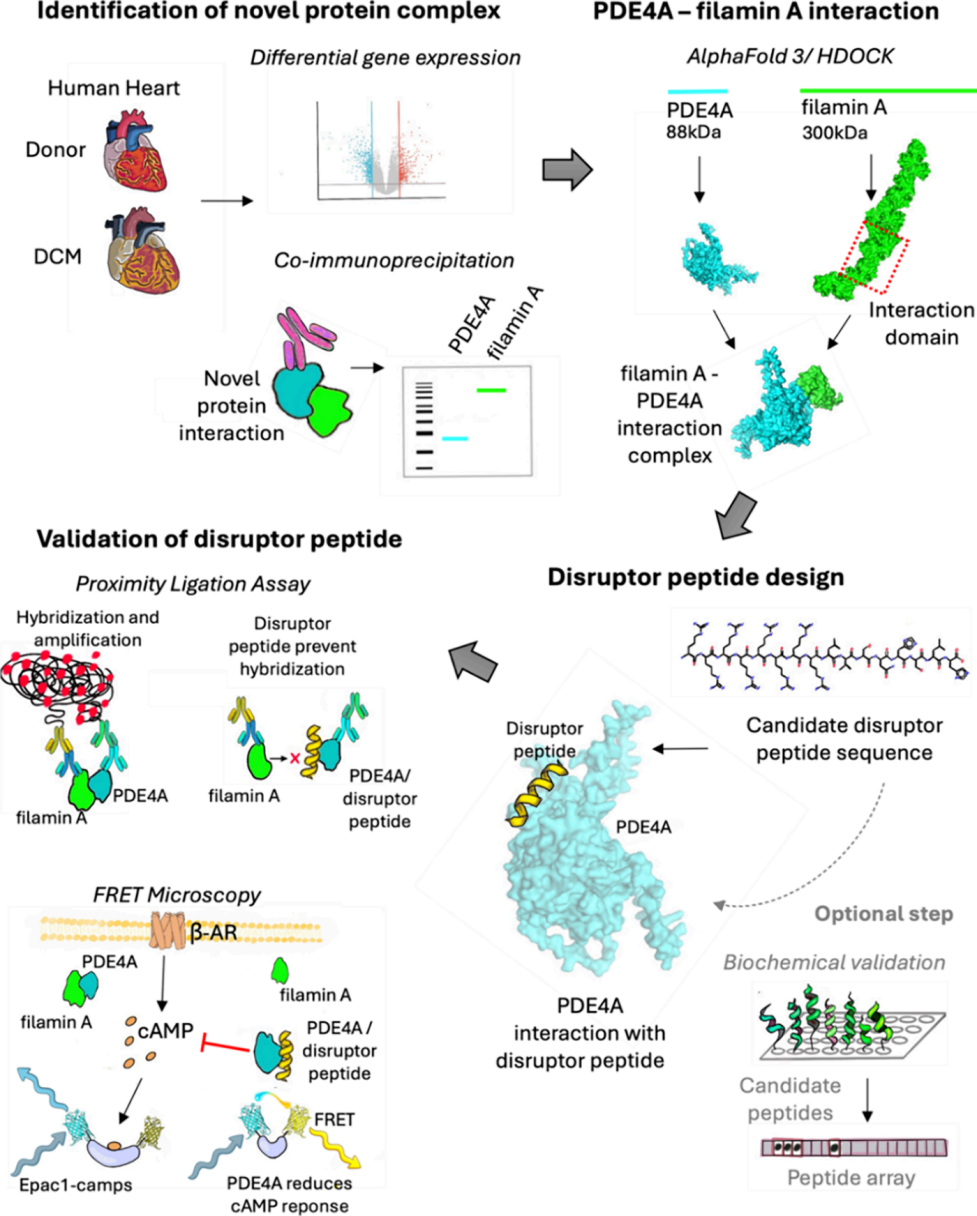
Schematic overview of the study design. Identification of a novel protein complex in the human heart affected by dilated cardiomyopathy (DCM) using differential gene expression of the scRNaseq data set and confirmation using coimmunoprecipitation. Bioinformatic approach of PDE4A and filamin A interaction modeling using AlphaFold3 and HDOCK. Design of a disruptor peptide based on the filamin A sequence, and its interaction with PDE4A. Validation of disruptor peptide using proximity ligation assay and FRET microscopy.

We identified a Filamin A peptide in PDE4 immunoprecipitants using mass spectrometry (Supplementary Table 1). This suggests a novel protein complex formed by PDE4A–filamin A in the heart tissue that has never previously been reported.

To determine whether expression of filamin A and PDE4A4 is changed during disease pathology, we compared scRNaseq data sets from donor and DCM patients.^16^ Only PDE4A expression was significantly reduced in DCM cardiomyocytes compared with donor controls (Figure 2a), while filamin A expression was unchanged (Figure 2b). The trend was preserved during DCM genotypic stratification (Supplementary Figure 1a,b). Pull-down assay confirmed the presence of PDE4A–filamin A complexes in the heart tissues from donors and DCM samples (Figure 2c, d).

**Figure 2:**
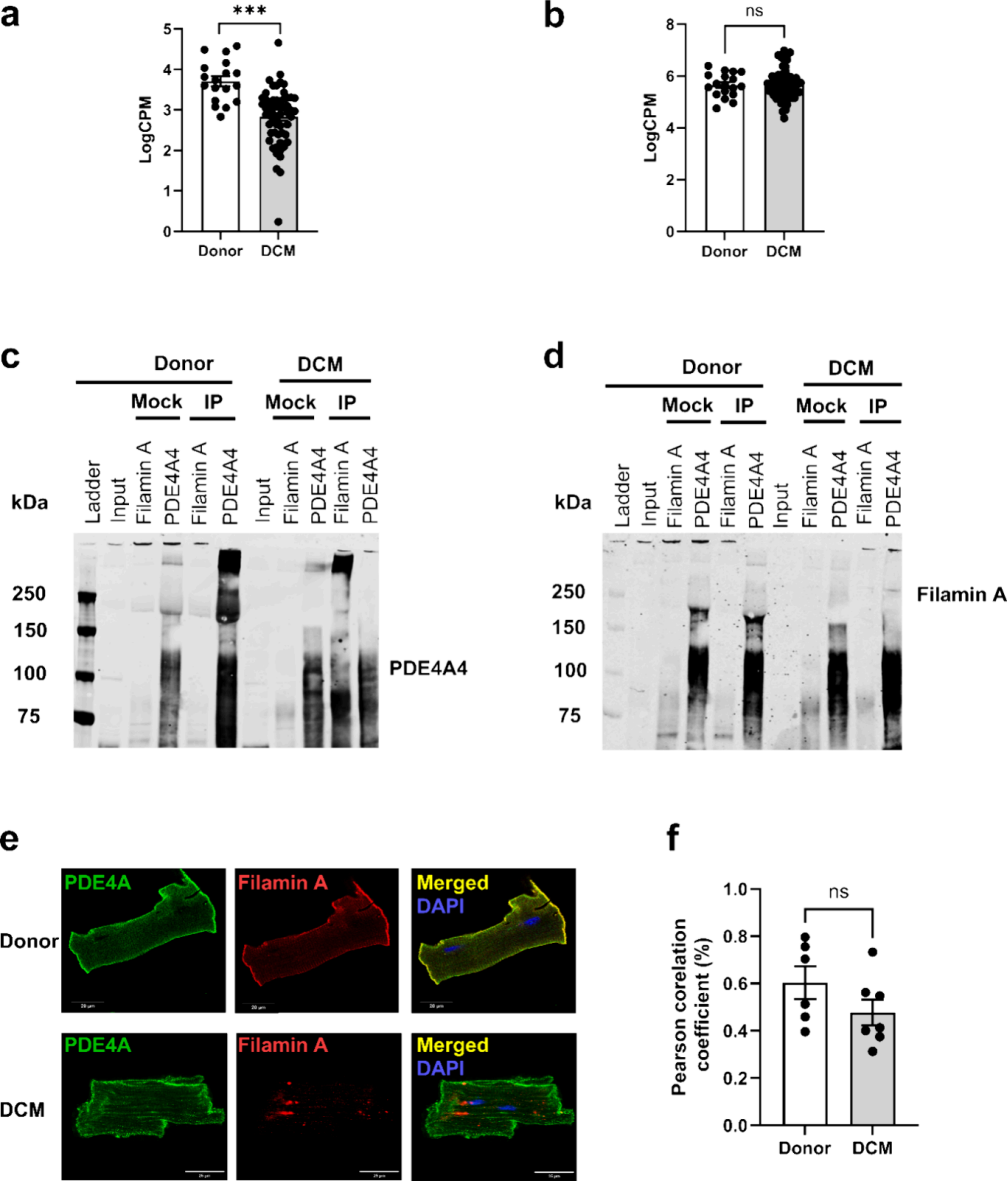
PDE4A and filamin A form a complex in human cardiomyocytes that is altered in heart failure. PDE4A (a) and filamin A (b) expression in DCM and donor heart based on single nuclei RNaseq, Bars indicate mean ± SEM, and dots represent individual donor samples, *N* = 18–58 (donor: 18, DCM: 58), two-sided Welch’s *t* tests. PDE4A expression was significantly reduced in DCM cardiomyocytes (***P < 0.001), whereas filamin A expression was not significantly changed (ns, *P* = [0.9759]). Human heart tissue were immunoprecipitated by PDE4A4 and probed for PDE4A4 (c) and filamin A (d). Input lanes represent input lysate of human left ventricular tissue and beads-only controls for the ‘mock IP’ lanes. (e) Representative immunostaining of PDE4A (green) and filamin A (red) in left ventricular cardiomyocytes from donor and DCM hearts. (f) Pearson’s correlation coefficient analysis of filamin A and PDE4A4 fluorescence signals in donor and DCM cardiomyocytes. *N* = 2, *n* = 6–7, Mann–Whitney test; ns, not significant, *P* = [0.2343].

Finally, there was a trend for Pearson coefficient of filamin A and PDE4A to be higher in donor cardiomyocytes (Figure 2e). Immunostaining analysis of isolated human cardiomyocytes revealed a membrane-localized distribution of the signals and a reduced spatial correlation between PDE4A and filamin A in DCM cells compared with donor cells, consistent with altered organization of the complex in disease (Figure 2e,f, Supplementary Figure 1c,d).

We established a multistep computational pipeline integrating AlphaFold3, and HDOCK to guide structure-based identification of a PDE4A–filamin A docking interface suitable for development into a disruptor peptide (Figure 3a).^24–28^ This workflow was designed to combine complementary prediction approaches with experimental peptide array validation to enable systematic nomination and refinement of candidate interface peptides.

**Figure 3:**
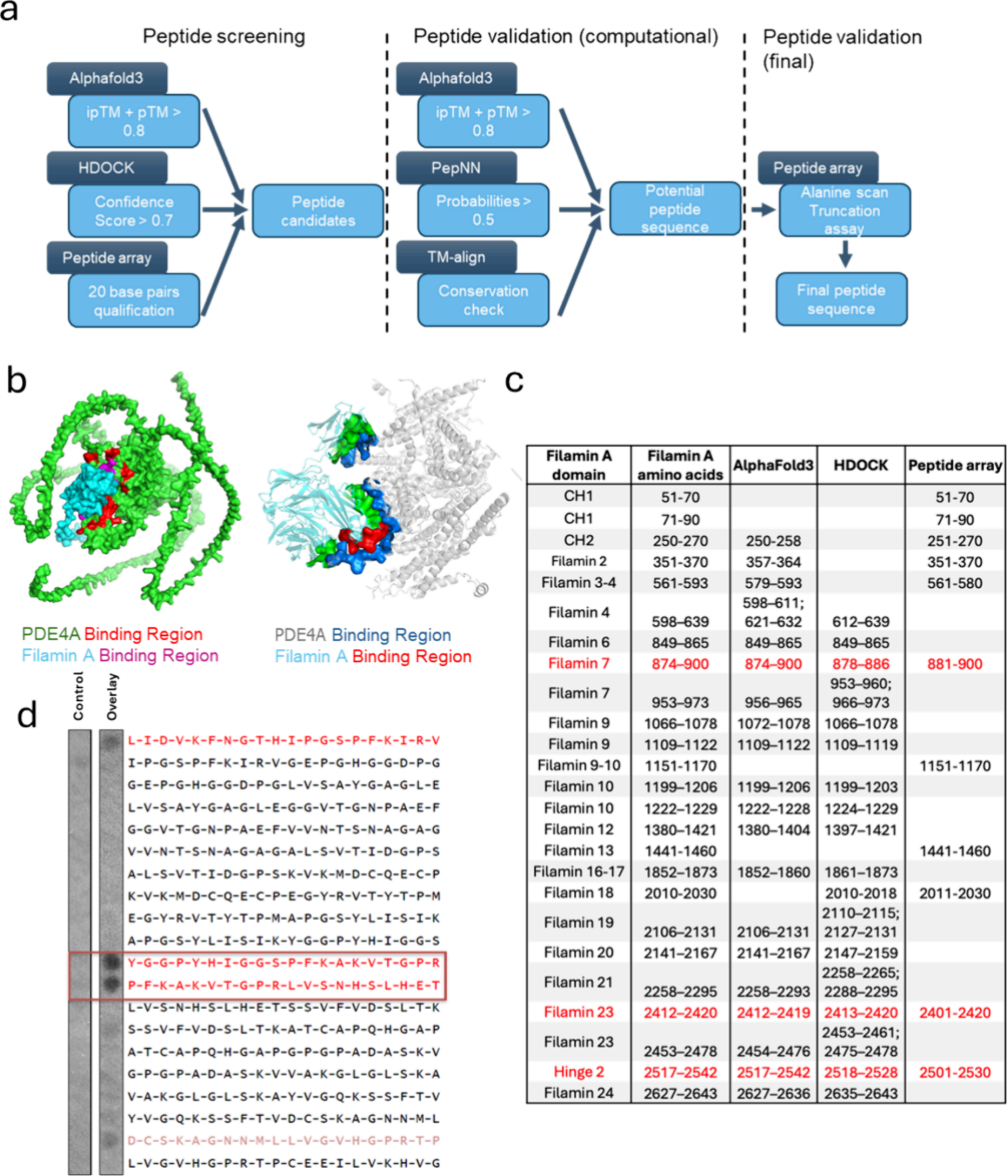
Computational identification and biochemical validation of a PDE4A–filamin A disruptor peptide. (a) Schematic overview of the multistep pipeline integrating computational (AlphaFold3 and HDOCK) and biochemical approaches to guide disruptor peptide identification and modification. (b) Predicted PDE4A binding regions (BR) to filamin A derived from AlphaFold3 (left) and HDOCK (right) modeling. (c) Peptide array screening and analysis identifying a minimal filamin A–derived peptide sequence capable of binding PDE4A. (d) The summary combines both computational prediction and peptide array results. The overlapped region between the three methods is labeled in red.

The pipeline was comprised of three sequential phases: (i) peptide screening, (ii) peptide validation, and (iii) peptide refinement. In the screening phase, putative PDE4A–filamin A interaction regions were first nominated using AlphaFold3 and HDOCK, while an unbiased peptide array screen was performed in parallel using overlapping filamin A-derived peptides (20 amino acids in length) probed with PDE4A (Figure 3b,c). These complementary approaches generated an initial set of peptide candidates. AlphaFold3 and HDOCK representative models identified overlapping PDE4A–filamin A interaction surfaces (Figure 3b), and integration with peptide array screening enabled prioritization of filamin A-derived candidate regions supported by both structural prediction and biochemical binding readout (Figure 3d).

During method development, we found that AlphaFold3 predictions became unstable when modeling large filamin A constructs, particularly full-length filamin A (aa 1–2647). Repeated AlphaFold3 runs followed by TM-align-based pairwise structural comparisons showed substantial variability across full-length models, with poor convergence reflected by high RMSD and low TM-scores (Supplementary Figure 2).^19,20^ In contrast, predictions performed on individual filamin A domains (or limited domain blocks) showed markedly improved structural consistency, including lower RMSD and higher TM-scores, both for filamin A structure prediction alone and for filamin A–PDE4A complex prediction (Supplementary Figure 2). Consistent with this, single-domain predictions typically achieved values compatible with better convergence (e.g., RMSD < 2 Å and TM-score >0.5), whereas full-length filamin A predictions were substantially less consistent (Supplementary Figure 2). Based on these observations, we adopted a domain-based modeling strategy for all subsequent AlphaFold3 and HDOCK analyses.

Using this optimized strategy, AlphaFold3 and HDOCK analysis were performed with individual filamin A domains as inputs against PDE4A, and candidate interaction segments were summarized alongside peptide array hits (Figure 3d). Cross-method comparison identified multiple candidate regions, including three filamin A-derived candidates that were supported by all three approaches (AlphaFold3, HDOCK, and peptide array screening), and these were advanced for further validation (Figure 3d). In the validation phase, the shortlisted candidate peptides were further assessed by peptide-centric structural modeling and peptide-binding pocket prediction (Figure 3a). AlphaFold3 was used to predict how each candidate peptide (10 peptide sequence from peptide array +3 overlapped peptide candidates) may engage PDE4A (Figure 4a). Analysis of AlphaFold3 predicted complexes further defined clusters of PDE4A residues predicted to contact filamin A, including regions encompassing S140–Y154 and additional interface segments (Figure 4a). These PDE4A interface regions were used as reference sites for subsequent peptide-binding analyses.

**Figure 4:**
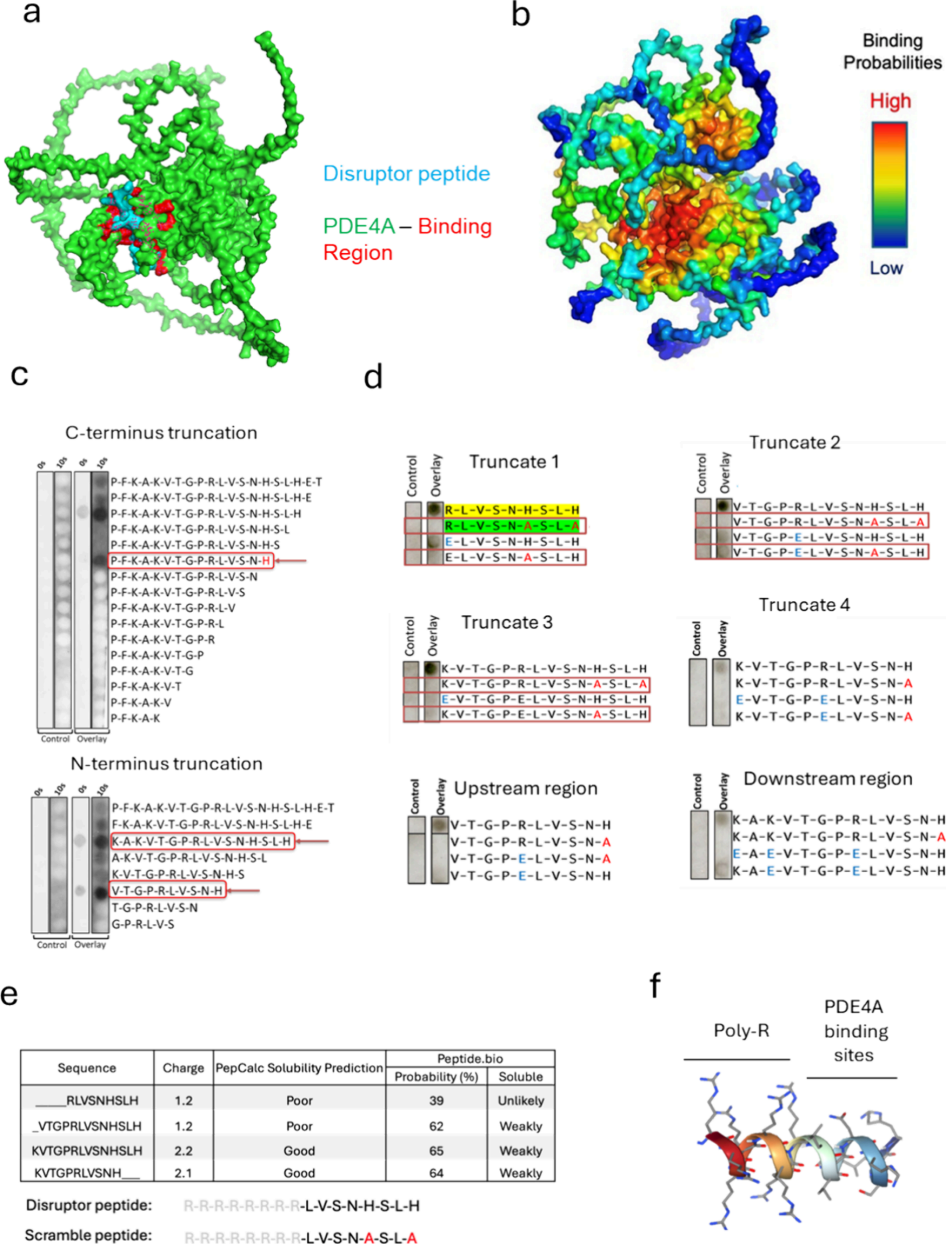
Design of the disruptor peptide targeting filamin A interaction region on PDE4A. (a) Predicted PDE4A binding regions (BR) to filamin A derived from AlphaFold3. (b) PepNN-based mapping of filamin A-derived peptide binding regions onto PDE4A. The red region indicates these regions from PDE4A have higher probability binding to filamin A-derived peptides. (c) Validation of the disruptor peptide sequence using truncation peptide array. N-terminus and C-terminus truncations peptide arrays were performed to determine the key residues for filamin A-PDE4A4 binding. (d) Alanine-scanning peptide array to identify residues required for PDE4A4 binding to filamin A. (e) Disruptor peptide optimization based on predicted physicochemical properties. Candidate sequences were evaluated for charge and solubility using PepCalc and Peptide.bio, leading to selection of a truncated peptide (RLVSNHSLH) modified with an N-terminal poly arginine tag for improved solubility. (f) 3D model featuring structure of disruptor peptide.

In parallel, PepNN was applied to map potential peptide-binding regions on PDE4A (Figure 4b).^29^ The filamin A sequence was segmented into overlapping 20-amino acid peptides, yielding 2628 candidate peptides, and screened against PDE4A. PepNN localized candidate peptides to defined binding pockets on PDE4A with a prominent region encompassing residues S140–Y154 (Supplementary Figure 4). However, PepNN did not provide robust ranking of individual peptide affinities and was therefore used for regional mapping rather than peptide prioritization. Candidate peptides were prioritized when their predicted PDE4A interaction regions overlapped with PepNN-predicted peptide-binding pockets in PDE4A (Figure 4a,b, Supplementary Table 2).

In the refinement phase, peptide array-based truncation and alanine-scanning analyses were performed to define the minimal active motif and identify residues critical for PDE4A binding (Figure 4c, 4d). Candidate interface peptides derived from the R2520-H2528 region were next evaluated experimentally using peptide array analysis (Figure 4c,d). Truncation and substitution analyses identified R2520–H2528 as the minimal sequence capable of binding PDE4A, and highlighted histidine residues H2525 and H2528 as critical contributors to the interaction, yielding disruptor and scramble peptide candidates (Figure 4c, d, Supplementary Figure 3).

To further improve peptide developability, candidate and optimized sequences were evaluated for predicted physicochemical properties, including charge and solubility, using PepCalc and Peptide.bio (Figure 4e). The final cell-permeant disruptor peptide consisted of an N-terminal poly arginine tag fused to the filamin A-derived PDE4A–binding motif RLVSNHSLH. The scramble peptide contained the same N-terminal poly arginine tag but carried alanine substitutions at residues identified by alanine-scanning peptide array as important for PDE4A binding, yielding RLVSNASLA. Thus, the scramble peptide was designed to preserve the delivery tag and overall peptide format while reducing sequence-specific PDE4A–filamin A interface engagement. For clarity, we refer to RLVSNHSLH as the “disruptor peptide” and RLVSNASLA as the “scramble peptide” throughout the manuscript. Peptide’s helical configuration was modeled structurally (Figure 4f).

Together, these results establish a robust stepwise workflow in which computational prediction (AlphaFold3, HDOCK, PepNN, TM-align) and peptide array screening/refinement converge to identify and optimize a filamin A-derived peptide predicted to disrupt the PDE4A–filamin A interaction (Figures 3 and 4).

To assess the cross-species applicability of the disruptor peptide, sequence and structural conservation of PDE4A between human and rat were analyzed using Clustal Omega and HHPred.^30,31^ Sequence alignment revealed a high degree of conservation across PDE4A (81% amino acid identity by BLASTp), including the region corresponding to the predicted disruptor peptide binding site (Figure 5a).

**Figure 5:**
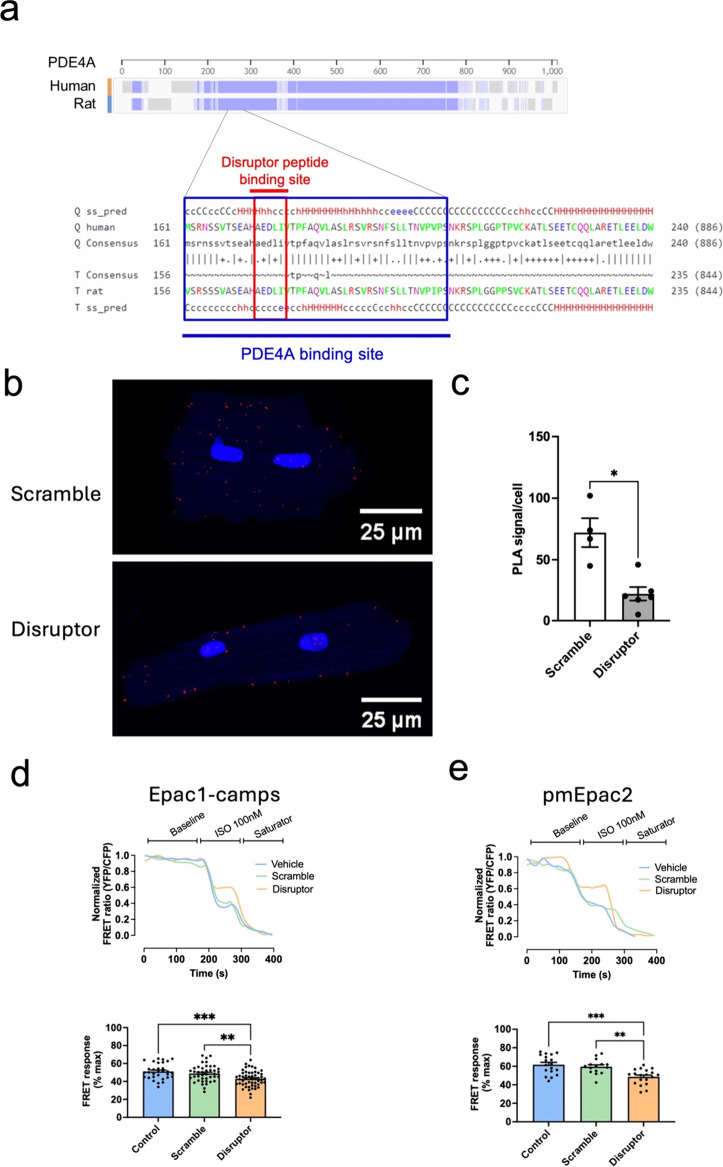
Disruptor peptide reduces PDE4A–filamin A proximity and attenuates cAMP response in rat cardiomyocytes. (a) The disruptor peptide binding region on PDE4A is similar between rat and human. PDE4A amino acid sequence Identities: 81%, calculated by BLASTp. Blue box: PDE4A–filamin A interaction region. Red box: Peptide binding region. (b) Representative proximity ligation assay images showing PDE4A–filamin A proximity treated with scramble peptide or disruptor peptide. PLA signal (green) and nuclei (DAPI, blue) are shown. (c) Quantification of PLA signal demonstrated a significant reduction in disruptor peptide-treated cardiomyocytes. *N* = 3, *n* = 4–6, Mann–Whitney test. Normalized FRET traces and normalized percentage maximum FRET ratio change measure with Epac1-camps (d) and pmEpac (e) FRET reporters. Data presented as mean± SEM *n* = 15–53 from 3 to 5 animals. *P < 0.05, **P < 0.01, ***P < 0.001 based on one-way ANOVA with Tukey’s multiple comparisons.

Structural superposition of human and rat PDE4A models demonstrated strong overall structural similarity (Figure 5a), which was quantified using TM-align (TM-score = 0.7532), yielding a structural similarity of 80.65% (Figure 5a). Together, these data indicate that both the sequence and structural context of the disruptor peptide binding region on PDE4A are conserved between species.

To determine whether the disruptor peptide alters the spatial association between PDE4A and filamin A in rat cardiomyocytes, PLA was performed. Cardiomyocytes treated with the disruptor peptide exhibited a marked reduction in PLA puncta compared with scramble-treated controls, indicating decreased close-range proximity between PDE4A and filamin A (Figure 5b). Quantification confirmed a significant reduction in PLA signal following disruptor peptide treatment (Figure 5c).

To determine whether disruption of the PDE4A–filamin A complex alters β-adrenergic cAMP signaling, cytosolic and membrane-associated cAMP dynamics were monitored in isolated rat cardiomyocytes using FRET-based sensors. Cytosolic cAMP responses were measured using the Epac1-camps sensor following stimulation with isoproterenol (100 nM). Cardiomyocytes treated with the disruptor peptide exhibited a significantly reduced normalized FRET response compared with vehicle and scramble peptides, indicating decreased cytosolic cAMP accumulation (Figure 5d).

To assess membrane-associated cAMP signaling, cardiomyocytes expressing the plasma membrane-targeted pmEpac2-camps sensor were analyzed. Disruptor peptide treatment resulted in a significant reduction in membrane-associated cAMP responses following β-adrenergic stimulation relative to controls (Figure 5e).

Together, these data demonstrate that selective disruption of the PDE4A–filamin A interaction attenuates both cytosolic and membrane-localized β-adrenergic cAMP signaling in rat cardiomyocytes, suggesting a displacement of functional PDE4A from filamin A complex.

We next determined whether the disruptor peptide alters the spatial association between PDE4A and filamin A in human donor cardiomyocytes. Human cardiomyocytes treated with disruptor peptide exhibited a marked reduction in PLA puncta compared with scramble-treated controls (Figure 6a). Quantification confirmed a significant reduction in PLA signal following disruptor peptide treatment in human donor cardiomyocytes (Figure 6b).

**Figure 6:**
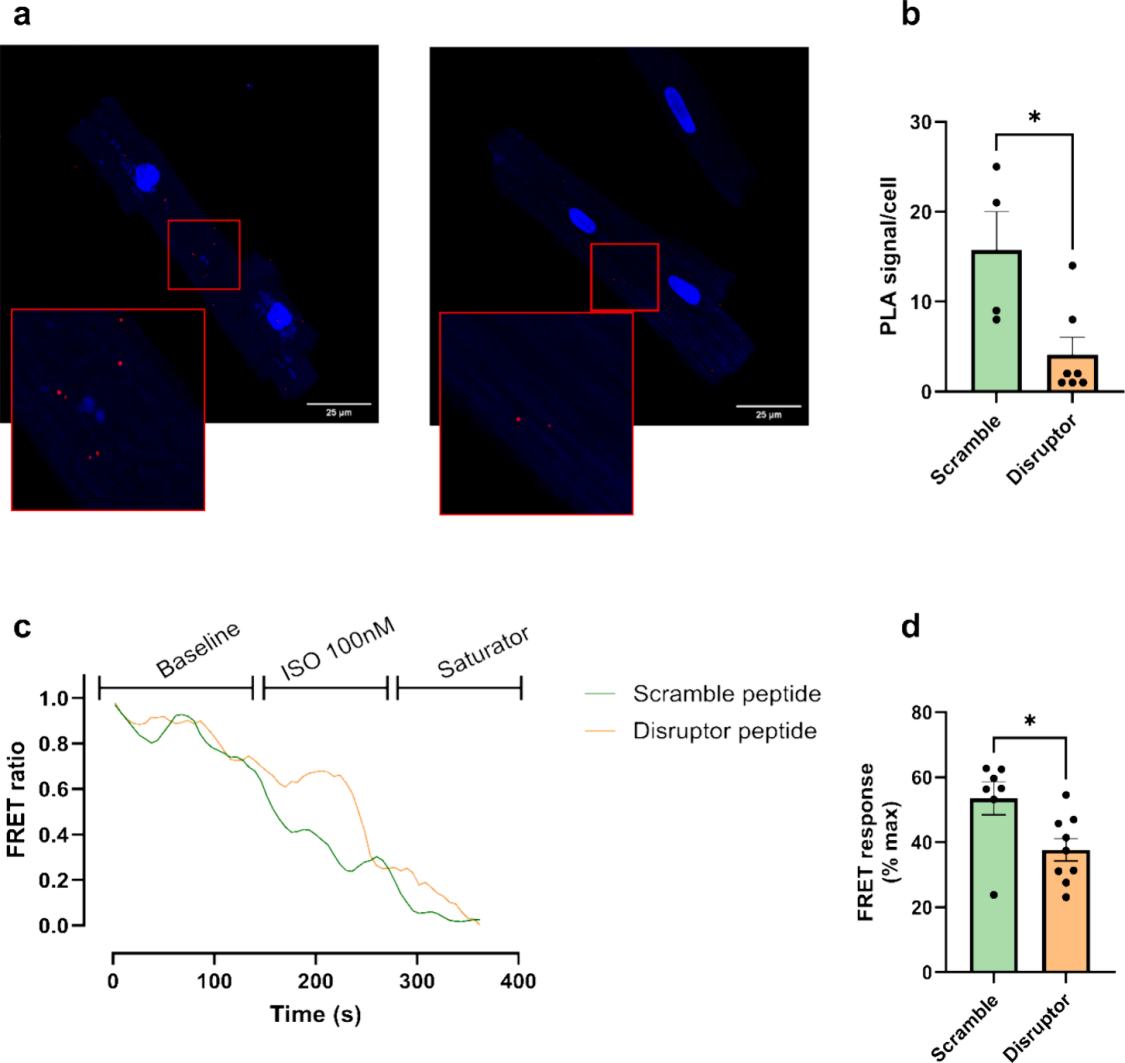
Disruptor peptide reduces PDE4A - filamin A proximity and attenuates cAMP response in human donor cardiomyocytes. Representative proximity ligation assay images (a) and quantification (b) showing PDE4A - filamin A proximity treated with scramble peptide or disruptor peptide. PLA signal (red) and nuclei (DAPI, blue) are shown. Quantification of PLA signal demonstrated a significant reduction in disruptor peptide–treated cardiomyocytes. *N* = 3, *n* = 4–7, Mann–Whitney test, *p < 0.05. Representative normalized FRET traces (c) and normalized percentage maximum FRET ratio change (d) measured with Epac1-camps FRET reporters. *N* = 4, *n* = 7–10, *p < 0.05, based on Welch’s *t* test. Data presented as mean ± SEM.

To determine whether disruption of the PDE4A–filamin A complex alters cytosolic β-adrenergic cAMP signaling in isolated human cardiomyocytes, Epac1-camps reporter was employed. Cardiomyocytes treated with the disruptor peptide exhibited a significantly reduced normalized FRET response compared with scramble controls, indicating decreased cytosolic cAMP accumulation (Figure 6c,d).

Together, these data demonstrate that selective disruption of the PDE4A–filamin A interaction attenuates both proximity of target proteins and cytosolic and β-adrenergic cAMP signaling in human donor cardiomyocytes.

Protein–protein interactions within large scaffold complexes represent critical regulators of spatially organized signaling, yet they remain largely inaccessible to traditional pharmacological strategies. Conventional drug discovery has focused predominantly on catalytic domains, receptors, or well-defined ligand-binding pockets.^41^ In contrast, multidomain scaffold proteins often lack discrete small-molecule binding sites, and their extended, dynamic interaction interfaces are considered functionally essential but structurally intractable.^42^ To address this challenge, we developed an integrated computational-experimental framework that enables systematic prediction of interaction interfaces, regional peptide mapping, and rational generation of cell-permeable disruptor peptides. Importantly, our data supports a PPI-directed mechanism of spatial signaling modulation rather than a conventional catalytic-site inhibitor mechanism. However, because bulk PDE4A enzymatic activity was not directly measured in this study, we have avoided concluding that global PDE4A catalytic activity is unchanged. Instead, the present findings suggest based on PLA results that targeting a predicted scaffold interface reduces PDE4A–filamin A interaction resulting in modulation of cAMP nanodomain compartmentalization supported by FRET results.

In this study, we developed and validated a convergent experimental-computational workflow to selectively disrupt the PDE4A–filamin A interaction in cardiomyocytes, enabling functional assessment of a scaffolding complex potentially implicated in maladaptive heart failure.^13^ The motivation is clinically compelling: heart failure remains a global health burden with high mortality and cost, and DCM represents a substantial and genetically complex contributor to this syndrome.^2,43^ Building on the concept that localized cyclic nucleotide microdomains shape cardiomyocyte responses and undergo disease-associated remodeling, we combined human tissue biochemistry, a convergent computational pipeline, and functional live-cell measurements to generate a cell-permeable disruptor peptide that decouples PDE4A from filamin A.^5^

A key methodological contribution of this work is the establishment of a reproducible closed-loop workflow: (1) computational prediction, (2) experimental array screening, (3) iterative optimization and its implementation for large multidomain scaffold proteins. Initial attempts to model the PDE4A–filamin A complex using full-length filamin A and PDE4A sequences in AlphaFold3 resulted in poor convergence and substantial variability between independent prediction runs, as confirmed by TM-align comparisons. This highlights a limitation of current structure prediction approaches when applied to elongated and highly flexible scaffold proteins. By decomposing filamin A into individual domains and performing domain-level interaction predictions, model consistency (RMSD score) and both pTM and ipTM confidence metrics were markedly improved. This domain-based strategy enabled more reliable nomination of candidate interaction interfaces and provided a tractable framework for subsequent experimental validation.

To avoid reliance on a single predictor, we further introduced HDOCK as an orthogonal validation tool to independently redock and cross-check the interfaces nominated by AlphaFold3. Candidates contact residues and surface regions were compared under a unified interface criterion (e.g., a 5 Å distance threshold). By running “domain-resolved AlphaFold3 + HDOCK” in parallel, we narrowed the candidate interaction region to higher-confidence interface segments that are potentially amenable to peptide targeting, thereby providing structural templates for subsequent peptide design.

To bridge protein–protein interface prediction with peptide-scale targeting, we applied PepNN as a peptide-binding site mapping tool on the PDE4A surface. We generated a library of overlapping 20-amino acid filamin A-derived peptides (one-residue sliding window) and used PepNN to predict, for each peptide, residue-wise binding probabilities on PDE4A. Across this systematic screen, PDE4A displayed 7 reproducible ‘hot’ regions with elevated propensity (>50%) to engage peptides, whereas differences between individual filamin A peptides at a given PDE4A site were comparatively modest. PepNN predictions successfully mapped these peptides to potential binding pockets on PDE4A; however, the algorithm did not robustly discriminate among candidate peptides in terms of relative binding likelihood or affinity ranking. Thus, PepNN was used primarily as a regional mapping tool rather than as a direct peptide prioritization method. By intersecting these PepNN-defined pockets with the PDE4A–filamin A PPI interface nominated by domain-resolved AlphaFold3/HDOCK modeling and with peptide-array binding/truncation data, we prioritized a filamin A motif most likely to overlap the native interface and thereby act as a disruptor of the PDE4A–filamin A interaction. Together, these observations underscore the importance of an iterative and hierarchical computational strategy that integrates domain-level structural prediction with peptide-level regional screening to guide experimental disruptor peptide design for large protein–protein interfaces.

A key finding is that PDE4A and filamin A form complexes in human myocardium. The Pearson coefficient between PDE4A and filamin A signals in isolated cardiomyocytes showed reduced trend, consistent with disease-associated reorganization of the complex. This aligns with the broader idea that heart failure is associated with remodeling of compartmentalized cyclic nucleotide control systems rather than uniform changes in total signaling output.^5^ Given the emerging importance of deep molecular phenotyping for identifying disease-linked pathways and networks, the observation that a specific PDE4A–cytoskeletal complex is reorganized in DCM supports a model where subcellular signaling topology contributes to pathological signaling.^44^ We demonstrate a practical, reproducible strategy for mapping and targeting a protein–protein interface in a large, multidomain cytoskeletal scaffold. Full-length AlphaFold3 complex predictions were initially nonconvergent, motivating a domain-based modeling strategy with consensus interface nomination. This decision is consistent with the strengths and known practical constraints of current structure prediction approaches for large multidomain assemblies and variable interfaces.^18^ By integrating AlphaFold3, orthogonal HDOCK docking, and PepNN, and then applying peptide arrays for biochemical validation, we identified a minimal filamin A-derived interface motif (R2520–H2528) required for PDE4A binding.^18,24,29^ We demonstrate a practical strategy for mapping and targeting a protein–protein interface in a large, multidomain protein system. Importantly, the workflow should be viewed as transferable rather than universally generalizable at this stage.

The designed peptide produced a clear molecular effect in cardiomyocytes: PLA signals between PDE4A and filamin A were reduced after treatment, indicating decreased close-range proximity without overt redistribution detectable by conventional confocal imaging. Such “functional uncoupling without gross relocalization” is consistent with a broader signaling principle: local second messenger responses depend on highly organized microdomains maintained by scaffolds, anchoring proteins, and spatially restricted effectors.^5,45^

Functionally, disrupting the PDE4A–filamin A interaction attenuated β-adrenergic cAMP responses in both cytosolic and plasma membrane-associated compartments. These data support a model in which PDE4A–filamin A contributes to selective amplification or maintenance of specific βAR-linked cAMP pools.^46^ This compartment-selective behavior is consistent with the broader framework that PDE isoforms and their anchoring complexes regulate discrete nanometre-scale cyclic nucleotide domains that control specific functional outputs.^5,47^

Filamin A is a mechanosensitive actin-binding scaffold that couples forces to binding partner selection and signaling outputs and is implicated broadly in cardiovascular remodeling pathways.^11,13^ At the molecular level, kinase accessibility to filamin A can depend on its global conformation and ligand engagement, not simply exposure of a phosphorylation motif, providing a plausible structural basis for how disease-associated cytoskeletal remodeling or altered binding partner occupancy could reshape local PDE/PKA signaling without changing global pathways.^12^

From a translational perspective, these findings reinforce the concept that targeting scaffolding interactions may offer a route to tune pathological signaling while sparing essential physiological outputs. AKAP-organized signalosomes are a precedent for this approach, where selective PPI disruption can rewire signaling microdomains with higher specificity than catalytic inhibition.^9,45^ In heart failure, where cyclic nucleotide signaling is extensively remodeled and PDE-directed therapies have shown mixed efficacy depending on isoform and context.^48^

Several limitations and next steps follow directly from our results. First, while PLA and peptide arrays indicate reduced PDE4A–filamin A engagement, the extent of disruption and the stoichiometry within native cardiomyocyte microdomains remain to be quantified. Second, our present functional readouts indicate reduced membrane and cytosolic cAMP transients, yet because there is no filamin A localized FRET reporter, we could not confirm associated increased localized cAMP in filamin A compartment. Given the conformation-sensitive nature of filamin A phosphorylation and ligand binding, future work should also assess whether disease-associated mechanical loading or cytoskeletal remodeling shifts peptide efficacy or interface accessibility.^12^ Third, although cross-species sequence/structure conservation supports applicability in rat experiments, the observed variability across repeated AlphaFold3 predictions underscores the importance of consensus modeling and experimental validation in peptide discovery workflows.^18^ Finally, extension to broader human cohorts and integration with emerging plasma/network biomarkers of HF development may aid in positioning this pathway within the multisystem biology of HF.^44^

In conclusion, we present a computational-experimental pipeline that nominates and validates a minimal interface disruptor peptide capable of selectively disrupting the PDE4A–filamin A complex in cardiomyocytes. Our functional data support a role for this complex in shaping compartmentalized βAR cAMP signaling, highlighting PPI-directed modulation of signaling microdomains as a promising strategy for mechanistic dissection and, potentially, therapeutic development in heart failure and DCM.^2,5,43,49^ Furthermore, the computational pipeline is potentially not limited to the filamin A–PDE4A complex and may be transferable to the design of disruptor peptides for other PDE protein–protein interactions.

## Supplementary Material



## Data Availability

All other data is available from the authors upon reasonable request. Scripts have been implemented in Python and R, which are available on GitHub (https://github.com/pksrivas/Disruptive_peptide). The scRNaseq data was used from a publicly available open source https://cellxgene.cziscience.com/collections/e75342a8-0f3b-4ec5-8ee1-245a23e0f7cb/private.

## Notes

Mass spectrometry data, additional transcriptomic and colocalization analyses, AlphaFold3/TM-align benchmarking, peptide array mapping and validation, PepNN prediction results, and tables of predicted interaction regions (PDF)

## Abbreviations

βAR: β-adrenergic receptor
AC: adenylyl cyclase
cAMP: cyclic adenosine monophosphate
DCM: dilated cardiomyopathy
FRET: fluorescence resonance energy transfer
HDOCK: hierarchical protein–protein docking
HF: heart failure
PDE: phosphodiesterase
PDE4A: phosphodiesterase 4A
PKA: protein kinase A
PLA: proximity ligation assay
PPI: protein–protein interaction
RMSD: root-mean-square deviation
SEM: standard error of the mean
TM-score: template modeling score
