# Supplementary material for: Development of a Nanoscale Protein–Protein Mapping of PDE4 Interface-Disrupting Peptides
Source: Nano Lett. 2026 Jul 1;26(31):10188–98. doi: 10.1021/acs.nanolett.6c01869 (PMC13475046; doi:10.1021/acs.nanolett.6c01869)
Supplement: Supplementary file 1 [file nl6c01869_si_001.pdf]

Development of a Nanoscale Protein−Protein Mapping of PDE4 Interface-Disrupting Peptides

Sibo Lyu, Aleksandra Judina, Jiayue Ling, Thomas A Wright, Aditi Kulkarni, Jiarong Fu, Iyobel Kibreab, George Baillie, Prashant Kumar Srivastava,* and Julia Gorelik*

Methods

1.1 Ethics approval

Human donor hearts were obtained with approval from the NHS Research Ethics Committee (Ref: 16/LO/1568). DCM samples from patients with end-stage heart failure were obtained from the Brompton Harefield & NHLI Ethics Committee Biobank (REC: 09/H0504/104+5).

All animal procedures were conducted at Imperial College London in compliance with the UK Animals (Scientific Procedures) Act 1986, the EU Directive 2010/63/EU, and the National Institutes of Health Guide for the Care and Use of Laboratory Animals.

1.2 Human tissue protein extraction

Human heart tissues were weighed out without allowing them to thaw and transferred to universals. Chilled CHAPS lysis buffer (5 ml) (Hepes pH 7.4 (50 nᴍ), NaCl (100 mᴍ), CHAPS (1%), EDTA (1 mᴍ), glycerol (1%)) supplied with protease and phosphatase inhibitors into the universals. Tissues were chopped into small pieces using clean, small-bladed scissors. Then the complex was homogenized for four 10-second cycles, chilling on ice between each cycle. After the last homogenization, the mixture was rotated for 1 hour at 4 ºC. Supernatant was collected by

centrifugation for 30 minutes at 14,000 rpm at 4 °C. The supernatant was collected and stored at -80 ºC before further use.

1.3 Immunoprecipitation

1 mg of total protein was used for each experimental condition. PDE4A4 primary antibody (10 µl) was added to the lysate, and the complex was incubated at rotation for 3 hours at 4 ºC. As a negative control, rabbit IgG (Sigma-Aldrich, 12-370) (10 µl) was included to screen for non-specific binding. The results of immunocomplexes were captured using Pierce Protein G Magnetic beads (Thermo Scientific™,88847) (50 µl). The beads were pre-cleaned three times with lysis buffer (300 µl) using the magnetic rack to remove any residual buffer and salts. The cleaned beads were added to the immunocomplexes and rotated overnight at 4 ºC. The following day, the complex was washed five times with fresh lysis buffer using the magnetic rack. After the last wash, the immunoprecipitation products were eluted by adding SDS protein sample buffer (35 µl), followed by vortexing to separate the beads from the bound proteins. Then the bound proteins were denatured by heating at 95 ºC for 5 minutes. The proteins were separated by magnetic rack, and western blotting was performed to assess the contents of the immunocomplex. IP gel bands were separated under UV light for mass spectrometry analysis.

1.4 MS Analysis of PDE4 Immunoprecipitants.

1 mg of human heart LV tissues from donors and DCM patients (n=3) were lysed in CHAPs buffer supplemented with protease inhibitors (Complete protease inhibitor mixture tablets; Roche) and phosphatase inhibitors (PhosSTOP tablets; Roche). Tissue lysates were centrifuged (20,000 g, 10 min), and the supernatant was immunoprecipitated with PDE4 antibody for 2 h at 4 °C. PDE4 immunoprecipitates were separated by SDS-PAGE, and gel bands were excised and

subjected to in-gel digestion. Gel pieces were washed with ammonium bicarbonate (100 mM) followed by acetonitrile (50%)/ammonium bicarbonate (100 mM), reduced with DTT, alkylated with iodoacetamide, shrunk in acetonitrile, and dried in a vacuum centrifuge. Proteins were digested overnight at 37 °C with trypsin (Promega) in ammonium bicarbonate (25 mM). Peptides were extracted with formic acid (5%) and acetonitrile, dried, and resuspended in acetonitrile (5%)/formic acid (0.5%). Samples were analyzed by nanoflow LC-ESI-MS/MS on a Thermo Scientific RSLCnano system coupled to an Orbitrap Elite mass spectrometer. Protein identification was performed using the Mascot search engine (v2.6.2, Matrix Science) against the SwissProt human database.

1.5 Western blot

Protein lysates were prepared in RIPA buffer supplemented with phosphatase inhibitors (PhosSTOP, Roche, 04906845001), protease inhibitors (cOmplete Mini, Roche, 11836153001), and dithiothreitol (DTT). Protein concentration was determined by the BCA assay. Equal amounts of protein were mixed with LDS sample buffer, denatured at 95 °C for 10 min, separated on SDS–PAGE gels (4-12%), and transferred to nitrocellulose membranes using a Xcell® SureLock MiniCell system (ThermoFisher Scientific, Paisley, UK). Membranes were blocked in 5% phosphoblocker (Cambridge Bioscience, Cambridge, UK) in TBS-T (Tween-20 (0.1%)) for 1 h, and incubated overnight at 4 °C with mouse anti-filamin A (1:100; Proteintech) and rabbit anti-pan-PDE4A (1:1000; in-house). [1] Following washing, membranes were incubated with fluorophore-conjugated secondary antibodies (1:10,000) for 1 h at room temperature. Signals were detected using a Licor Odyssey scanner (Licor Odyssey, Nebraska, USA) Digital images were collected and analyzed using Licor Odyssey software (Licor Odyssey, Nebraska, USA).

1.6 Single-cell RNA-seq pseudobulk analysis of PDE4A and FLNA expression in cardiomyocytes

Single-nucleus RNA-seq data from Reichart et al. were obtained from human ventricular myocardium (full-thickness left ventricular free wall) from non-failing donor controls and failing non-ischemic cardiomyopathy hearts, and were generated using 10x Genomics Chromium 3′ chemistry. [2] The analyzed cohort comprised 79 hearts in total (18 controls, 61 failing hearts); for subtype analyses, the principal DCM genotype groups included LMNA (n = 12), RBM20 (n = 8), TTN (n = 12) and PV-negative DCM (n = 8). Data were analyzed using a pseudobulk strategy at the donor level to assess changes in PDE4A and FLNA expression in cardiomyocytes. Initial processing (alignment/mapping, cell calling, and primary QC, cell type annotation) were performed by the original study. For donor-level inference, a pseudo-bulk count matrix was generated in Python (sciscRNA_edgeR.ipynb) by summing raw UMI counts across cells within each group. Pseudo-bulk samples were filtered by a minimum cell threshold of min_cells=100 per donor within cardiomyocytes. Counts were exported in the conventional edgeR orientation genes × samples.

For differential expression testing, pseudobulk counts were analyzed using edgeR (using quasi-likelihood model). [3] Expression is shown at the donor level as logCPM for PDE4A and FLNA. Donor-level logCPM values were exported as a gene expression donor-level matrix for downstream graphing (e.g., Prism). Statistical significance annotations shown on these plots (asterisks) were computed from donor-level logCPM values using edgeR glm tests. Asterisks were defined as: $P < 0.05$ (*), $P < 0.01$ (**), $P < 0.001$ (***).

1.7 In silico prediction of protein interaction sites

Where experimentally determined full-length structures were unavailable, three-dimensional models were generated using the web-based implementation of AlphaFold3. [4] Full-length amino acid sequences were submitted using default parameters. To improve prediction robustness and reduce structural complexity, segmented domains of human filamin A (UniProt: P21333-1) were evaluated for interaction with human PDE4A (UniProt: P27815-1). For each filamin A domain, AlphaFold3 was executed in three independent runs, generating five models per run (15 models per domain). Independent runs were performed to account for stochastic sampling and to assess structural convergence.

Structural similarity between predicted models and crystal structures was evaluated using TM-align in RCSB PDB. [5, 6]

- RMSD (root mean square deviation) quantifies the average distance between aligned Cα atoms after optimal superposition (Å), with lower values indicating closer structural agreement.
- TM score was used to assess global topological similarity between structures. [5]

Model confidence was evaluated using AlphaFold3 confidence metrics: [4]

- Predicted TM-score (pTM) to assess global structural reliability of each model.
- Interface predicted TM-score (ipTM) to evaluate the confidence of predicted inter-chain interface geometry.
- Per-residue confidence scores (pLDDT) to assess local structural stability and reliability of predicted interface residues.

Domains were prioritized for further analysis if at least three models demonstrated similar binding conformations and achieved pTM + ipTM > 0.8. The pTM metric reflects the predicted accuracy of the overall structural model, whereas ipTM measures the predicted accuracy of one component of the complex relative to its interacting partner. Structural convergence across independent predictions was evaluated by pairwise structural comparison and qualitative assessment of interface topology consistency.

1.8.1 Interface Definition and Consensus Mapping

Protein–protein interfaces were analyzed in PyMOL. Interface residues were defined as residues from either protein with any atom located within 5 Å of the binding partner. [7-9]

Candidate interaction regions were identified using a consensus-based framework integrating:

1. Reproducible interface regions across independent AlphaFold3 models.
2. Elevated ipTM values supporting interface confidence.
3. High pLDDT at the predicted interaction surface.
4. Surface accessibility and spatial clustering of residues visualized in PyMOL.
5. Evolutionary conservation across homologous sequences assessed using structural alignment approaches such as HHpred.

Residues were classified as consensus interface residues if they appeared within the defined 5 Å interaction boundary in at least three of the 15 models. These consensus regions were subsequently used as templates for disruptor peptide design.

1.8.2 Complementary Computational Validation

To further validate predicted interfaces, protein–protein docking was performed using the HDOCK server, which integrates template-based modeling with ab initio docking. [10-14] AlphaFold-predicted structures of filamin A and PDE4A were used as input. Homology-based model construction was performed using templates from the HDOCK protein–protein interaction database.

Docked complexes were analyzed in PyMOL, and interface residues were defined as residues with any atom located within 5.0 Å of the binding partner. [7, 9] Docking scores were calculated using the HDOCK knowledge-based iterative scoring functions ITScorePP (protein–protein) or ITScorePR (protein–peptide). Residue pairs within 5.0 Å between receptor and ligand were considered interface residues.

1.9 Peptide array synthesis

Peptide arrays spanning the full length of filamin A in 25mer segments with overlapping 5-amino acid shift, and those containing subsequent peptide binder libraries were prepared by SPOT synthesis using the Intavis Multipep RSi automated parallel peptide synthesizer. Polyethylene glycol-derivatized cellulose membranes (CEM) were used as a solid support, and peptides were synthesized from C to N terminus. DIC (N,N'- Diisopropylcarbodiimide, Sigma-Aldrich, cat. D125407) and Oxyma Pure (Ethyl 2- cyano-2-(hydroxyimino)acetate, Merck, cat. 8510860500) were applied to ‘activate’ Fmoc amino acids suspended in DMF (n,n-dimethylformamide, Rathburn, cat. RG2014) before spotting to the membrane support. Growing peptide chains were capped with acetic anhydride (Sigma- Aldrich, cat. 539996), before Fmoc deprotection using piperidine (Sigma-Aldrich, cat. 104094). The membrane was washed with

NMP (1-Methyl-2- pyrrolidinone, Sigma-Aldrich, cat. 443778). The spotting cycle was repeated until the 25mers were formed. Work-up of the membranes comprised incubation with deprotection solution (triisopropylsilane (2%) (Sigma-Aldrich, cat. 233781) and distilled water (5%) in trifluoroacetic acid (Fluorochem, cat. F008708)) at room temperature for two hours with gentle agitation. The membranes were then washed three times for 2 minutes each with DMF, DCM (Sigma-Aldrich, cat. 650463), then ethanol. Once dry, the arrays were inspected and cut to size using a transilluminator (Fisher Scientific, cat. 16515180), then stored at 4 °C.

1.10 Peptide array screening

Membranes were activated using absolute ethanol for 10 minutes and washed with TBS-T for 10 minutes. Membranes were then blocked in TBS-T containing BSA (5%) for 4 hours with constant agitation. Membranes were incubated with healthy human heart tissue lysate containing a total of protein in CHAPs buffer at 4 ºC overnight. On the following day, the membranes were washed 3 times with TBS-T for 10 minutes and incubated with PDE4A4 antibody (in-house) at 1 in 1000 for 3 hours at room temperature. After washing with TBS-T 3 times for 10 minutes, the membranes were incubated with anti-rabbit HRP conjugated secondary antibody (Sigma, NXA931) for 1 hour at room temperature before visualizing with ECL reagent via X-OMAT Medical film processor (KONICA MINOLTA,China). As a control, Filamin A membranes not overlaid with human heart tissue lysate also underwent the same primary and secondary antibody incubations as the experimental group. Alanine scan peptide array and truncation peptide array were also designed and performed in the same manner to identify the key amino acid residue for the PDE4A4-Filamin A binding.

1.11 Computational design of peptides targeting the PDE4A-filamin A interface

We aimed to design a cell-permeable peptide to selectively disrupt the PDE4A–filamin A complex without affecting PDE4A's catalytic activity, enabling functional dissection of its scaffolding role. Candidate peptides were nominated by integrating domain-resolved AlphaFold3 modeling, HDOCK protein–protein docking, and experimental peptide array screening

To map peptide-compatible pockets on PDE4A, PepNN was used as a residue-level peptide-binding site predictor. [15] Filamin A was segmented into overlapping 20-amino-acid peptides using a one-residue step.

Full-length human filamin A sequence (aa 1–2647; UniProt: P21333-1) was segmented into overlapping 20-amino acid peptides using a one-residue sliding window (2628 peptides). Each peptide sequence was evaluated against PDE4A (UniProt: P27815-1) to obtain per-residue probabilities on PDE4A indicating the likelihood that a residue participates in a peptide-binding site.

PepNN predicted residue-level binding probabilities for PDE4A structures from AlphaFold, with high-confidence binding domains identified using statistical modeling and multiple-testing correction (Python statsmodels). Annotated PDB files were visualized in PyMOL to guide peptide selection. HDOCK docking provided complementary predictions of peptide–protein binding modes and energetics, and the combined analysis across methods prioritized peptides with the highest predicted specificity and affinity.

1.12 Cross-species sequence and structural conservation analysis of peptide binding sites on PDE4A

To assess cross-species conservation of PDE4A human and rat protein sequences (UniProt: P27815 [PDE4A_HUMAN], P54748 [PDE4A_RAT]) were compared using Clustal Omega and HHpred to evaluate sequence identity, similarity, and conservation of predicted secondary structure elements. [16, 17]

To determine whether structural features were conserved in addition to primary sequence, three-dimensional models for human and rat proteins were obtained from the AlphaFold database (or generated using AlphaFold3 where required). Structural alignment between species was performed using TM-align to compare corresponding domains and evaluate cross-species structural conservation. Root-mean-square deviation (RMSD) and template modeling (TM) scores were calculated to quantify structural similarity and fold preservation.

Conservation of both amino acid sequence and overall three-dimensional architecture was used to assess the cross-species applicability and structural robustness of the designed disruptor peptides.

For the final peptide, there was a N-terminal R7 tag added for permeabilization. Peptides were ordered with N-acetylation and C-amidation for stability from the GenScript company. The alanine knockout peptide was labeled as scramble peptide. Peptide groups and sequences were listed: Disruptor (GenScript) RRRRRRRRLVSNHSLH, scramble peptide (GenScript) RRRRRRRRLVSNASLA, or vehicle (distilled water).

Hazard Statement: The peptide described here is a novel synthetic research peptide. No unexpected, unusual, or significant hazards were encountered during this work beyond those normally associated with the handling of synthetic peptides and routine biochemical reagents.

Accordingly, all experiments were performed using standard laboratory safety procedures and appropriate precautions for research chemicals.

1.13 Human cardiomyocyte isolation

Human donor left ventricular tissues unsuitable for clinical transplantation were obtained from UK transplant centers, and DCM tissue samples were collected from explanted heart during transplantation surgery. Tissues were flushed with cardioplegia solution and transported on ice-cold prior cell isolation. Ventricular cardiomyocytes were isolated using sequential enzyme solution digestion with protease and collagenase, following established protocol. [18, 19]

1.14 Rat cardiomyocyte isolation

The rat LV cardiomyocytes were isolated from adult male Sprague-Dawley rats (250-350 g; Charles River, UK) by Langendorff retrograde perfusion and enzymatic dissociation, as described previously. [20, 21]

1.15 LV cardiomyocyte culture

Isolated cells were plated on culture dishes, borosilicate glass coverslips (9 or 25 mm), or MatTek dishes pre-coated with laminin and maintained in serum-free minimum essential medium (MEM) before use in functional or biochemical assays. When applicable cells were incubated for 2h with designed disruptor (10 μᴍ), scramble peptide (10 μᴍ), or vehicle (distilled water).

1.16 Immunostaining of filamin A and PDE4A

Cardiomyocytes were incubated overnight at 4°C with mouse anti-filamin A (1:200) and rabbit anti-pan-PDE4A (1:200) primary antibodies diluted in blocking solution (goat serum (10%), BSA (5%), Tween-20 (0.2%) in PBS). Cells were then incubated with Alexa Fluor 488-conjugated anti-mouse and Alexa Fluor 546-conjugated anti-rabbit secondary antibodies (1:500) for 2 h at room temperature in the dark. Samples were mounted using ProLong Gold Antifade Mountant with DAPI (Vector Laboratories).

1.17 Confocal imaging and analysis

Fluorescence signal images were obtained using Leica Stellaris 8 Inverted Confocal Microscope using 63x 1.4 Numerical Aperture (NA) objective. Acquisition settings: smart gain: 20.0%, smart intensity: 30.0%, pinhole: 1.00AU, zoom: 1.28, Z-step size: 0.60μ m, Z-step number: 10. Signal colocalization was quantified using Pearson and Manders (M1 and M2) coefficients using the JACoP plugin in Fiji (ImageJ2, v. 2.9.0/1.80q). [22] For each image, the identical threshold values were applied to all images within the same experiment, as disruptor peptide, scramble peptide, and vehicle control samples were processed and imaged in parallel. Statistical significance was assessed by the Mann-Whitney test between donors and DCM patients.

1.18 Proximity ligation assay (PLA)

Cells were incubated overnight at 4 °C with mouse anti-filamin A (Proteintech) and rabbit anti-pan-PDE4A (in-house) primary antibodies diluted in Duolink® Antibody Diluent. Then PLA was conducted according to the Duolink® In Situ PLA kit (Sigma-Aldrich) manufacturer's instructions.

PLA signals were quantified using Fiji (ImageJ2) by counting discrete fluorescent puncta per cell. Individual cells were manually outlined based on DAPI staining, and the number of PLA puncta within each cell was measured using identical detection parameters across all conditions within the same experiment. Disruptor peptide-treated, or scramble peptide-treated control samples were processed, imaged, and analyzed in parallel, and identical threshold settings were applied to all groups. The average number of PLA puncta per cell was calculated for each condition and used for subsequent statistical analysis.

1.19 FRET microscopy

Rat LV cardiomyocytes were infected with adenoviral FRET vectors carrying Epac1-cAMP and pmEpac2 biosensors (100 MOI). [23-25] FRET ratio recordings were obtained using Multi-cell FRET microscopy previously described. [26] Cells expressing pmEpac2 or Epac1-camps were stimulated with isoprenaline (ISO) (100 nM). [21] Responses were normalized to saturating solution of IBMX (100 μM) and forskolin (10 μM). Statistical significance was assessed using the one-way ANOVA test with Tukey’s multiple comparisons (GraphPad Prism 10.6.1).

Supporting Information.

Supplementary Table 1 This table summarizes the filamin A peptide identified in PDE4 immunoprecipitates by mass spectrometry. It provides supporting biochemical evidence that filamin A is present in PDE4-associated complexes in human heart tissue.

**Filamin A; SwissProt_AC (Human heart tissue)**			
Peptide identified	Residues	Mass	Error (ppm)
VTAQGPGLEPSGNIANK	384-400	1651.85	1.30

Supplementary Table 1 | Filamin A peptide identified in PDE4 immunoprecipitation. The immunoprecipitation product of PDE4 gel bands was cut off and sent off for mass spectrometry analysis. Filamin A peptide was identified as a PDE4-associated protein, as listed.

Supplementary Figure 1 This figure provides additional transcriptomic and colocalization analyses supporting the relationship between PDE4A and filamin A in donor and DCM cardiomyocytes. It includes genotype-stratified RNA expression data together with Manders' coefficient analysis of PDE4A4 and filamin A immunostaining signals.

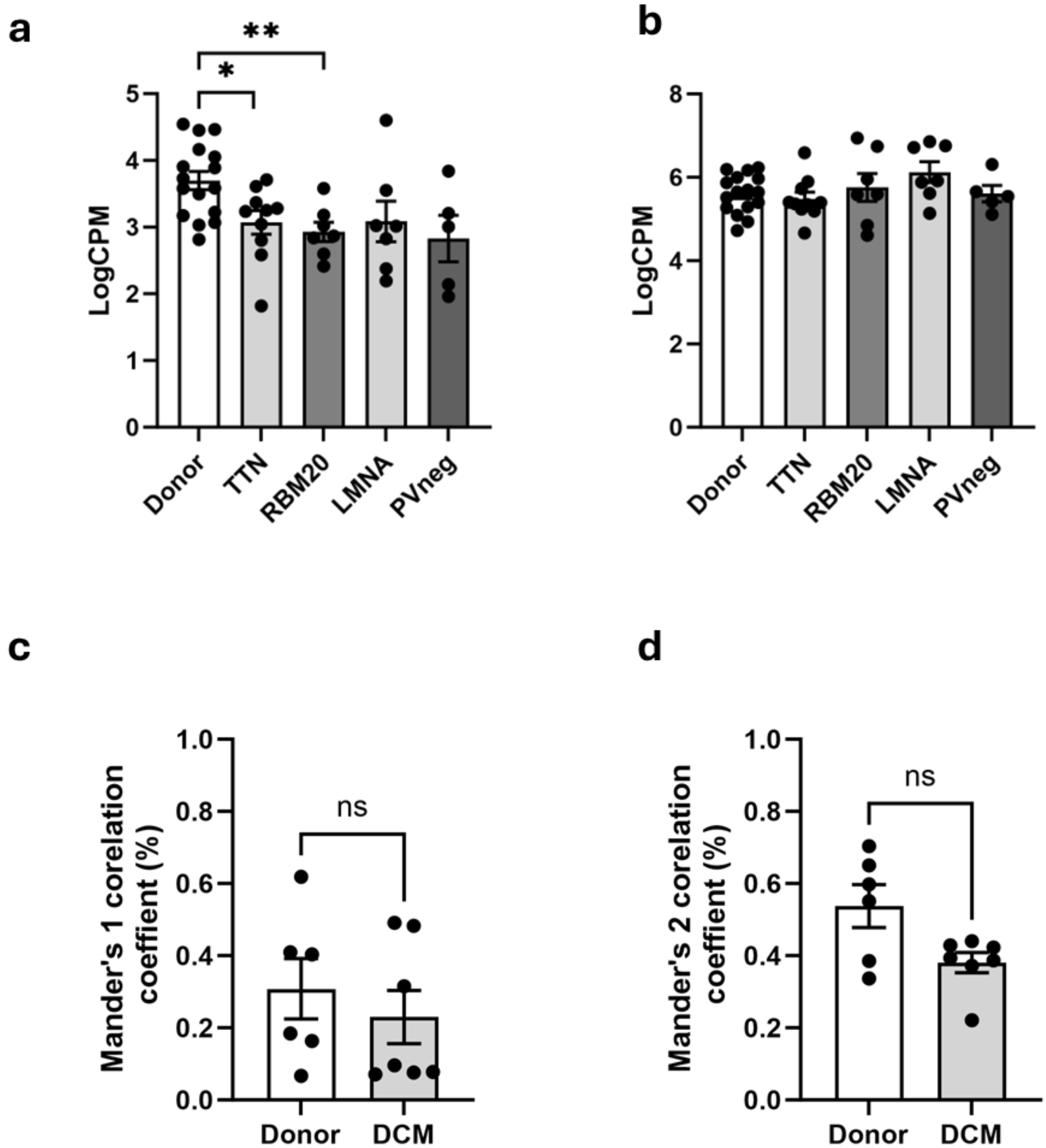

Supplementary Figure 1 PDE4A and filamin A RNA expression and levels in healthy and diseased human cardiomyocytes. (a) PDE4A and filamin A (b) expression in left ventricle from donor and across DCM genotypes based on scRNAseq. Bars indicate mean ± SEM, and dots represent individual donor samples. N=5-16 (Donor N:16, DCM patients with TTN mutation: 10, RBM20 mutation: 7, LMNA mutation: 7, PVneg: 5), Statistical significance annotations were

calculated from donor-level logCPM values using edgeR glm test. * Represents $P < 0.05$. Manders' correlation coefficient analysis of filamin A and PDE4A4 signal in human donor and DCM immunostaining. (c) Mander's 1 representing the overlap of filamin A fluorescent pixel signal with PDE4A4. [P=0.6282] (d) Mander's 2, representing PDE4A4 fluorescent pixel signal overlap with filamin A. [P=0.1807] Data presented as mean +/- SEM, n=6-7 from 2 patients, ns, no statistical significance was identified based on the t-test.

Supplementary Figure 2 This figure evaluates the stability and structural consistency of AlphaFold3 predictions for filamin A and filamin A–PDE4A complexes. It shows that full-length filamin A predictions were less consistent than domain-based models, supporting the use of a domain-resolved modeling strategy in the subsequent workflow.

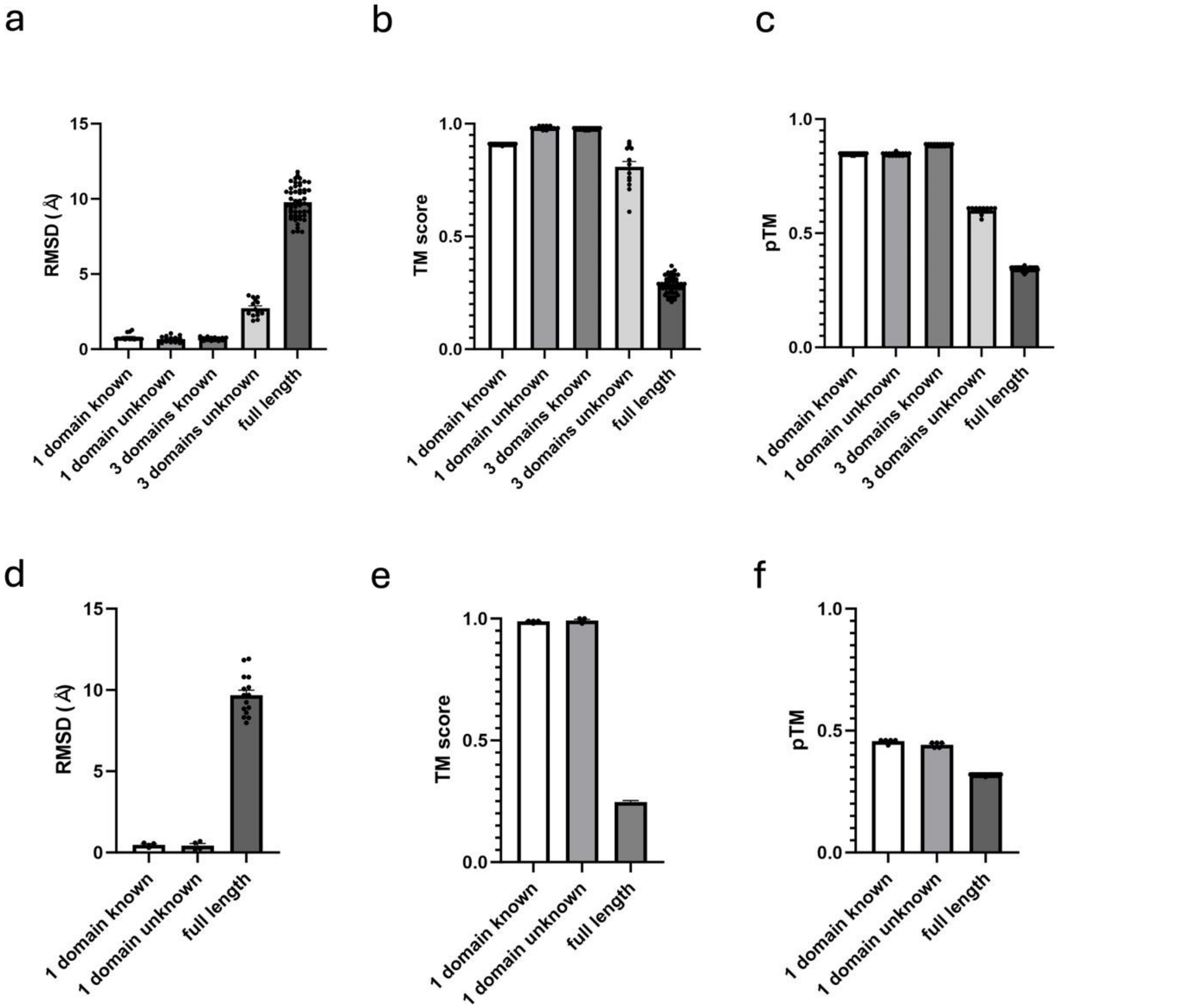

Supplementary Figure 2 | AlphaFold3 becomes unstable when predicting large proteins. Structure similarity of AlphaFold3 predicted filamin A protein using TM-align, giving RMSD (a), and TM score (b). The confidence score pTM generated from AlphaFold3 (filamin A) were listed in (c). The proteins are listed here: AlphaFold3 predicted 1 filamin A domain 21 with known crystal structure (9LWX), AlphaFold3 predicted filamin A domain 6 with unknown crystal structure, AlphaFold3 predicted filamin A 3 domains (domain 3, 4, 5) with known crystal structure (4M9P), AlphaFold3 predicted filamin A 3 domains (16, 17, 18) with unknown crystal structure, and AlphaFold3 predicted full length filamin A proteins (from aa 1-2647). Structure

similarity of AlphaFold3 predicted filamin A structures interaction with PDE4A proteins using TM-align (d, e). The confidence score pTM generated by AlphaFold3 (filamin A-PDE4A) was listed in (f). RMSD (root mean square deviation) and TM-score (template modeling score) were given from TM-align, pTM were generated by AlphaFold3.

Supplementary Figure 3 This figure maps the filamin A interaction region involved in PDE4A4 binding using peptide array–based validation. It includes peptide array screening, alanine scanning, and truncation analyses to define the minimal binding sequence and identify residues important for the interaction.

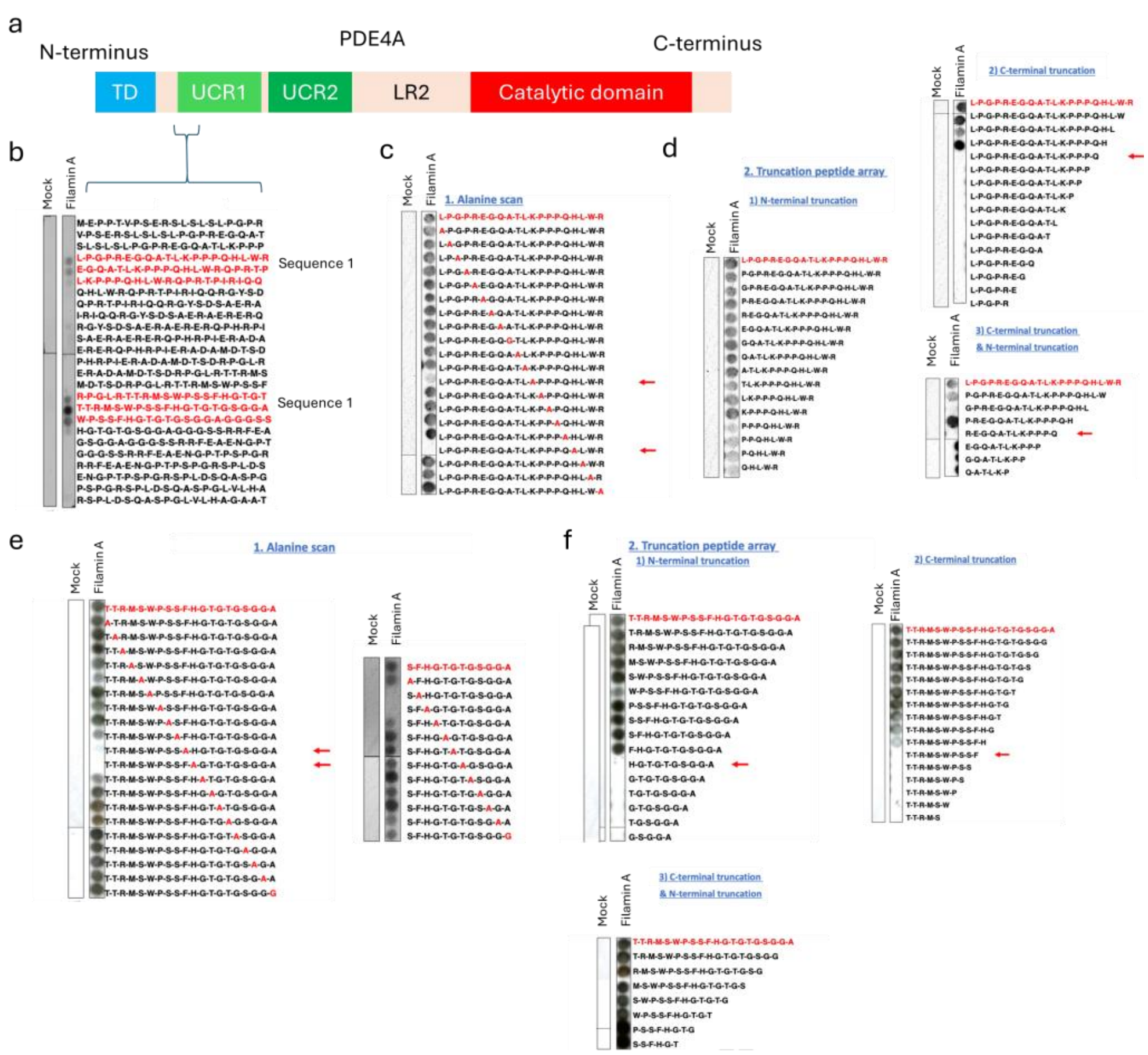

Supplementary Figure 3 | Mapping the filamin A docking region on PDE4A4 (a) Structure of PDE4A regions. (b) Peptide arrays containing the N-terminus of the PDE4A protein were overlain with lysate from donor tissue or Mock. Dark spots represent peptides bound to filamin A and identified two sequences. Sequence 1 (c, d) and sequence 2 (e, f) were tested using an alanine scan and a truncation peptide array.

Supplementary Figure 4 This figure presents PepNN-based prediction of PDE4A regions with high probability of binding filamin A-derived peptides. It provides complementary computational support for the localization of peptide-compatible binding regions on PDE4A.

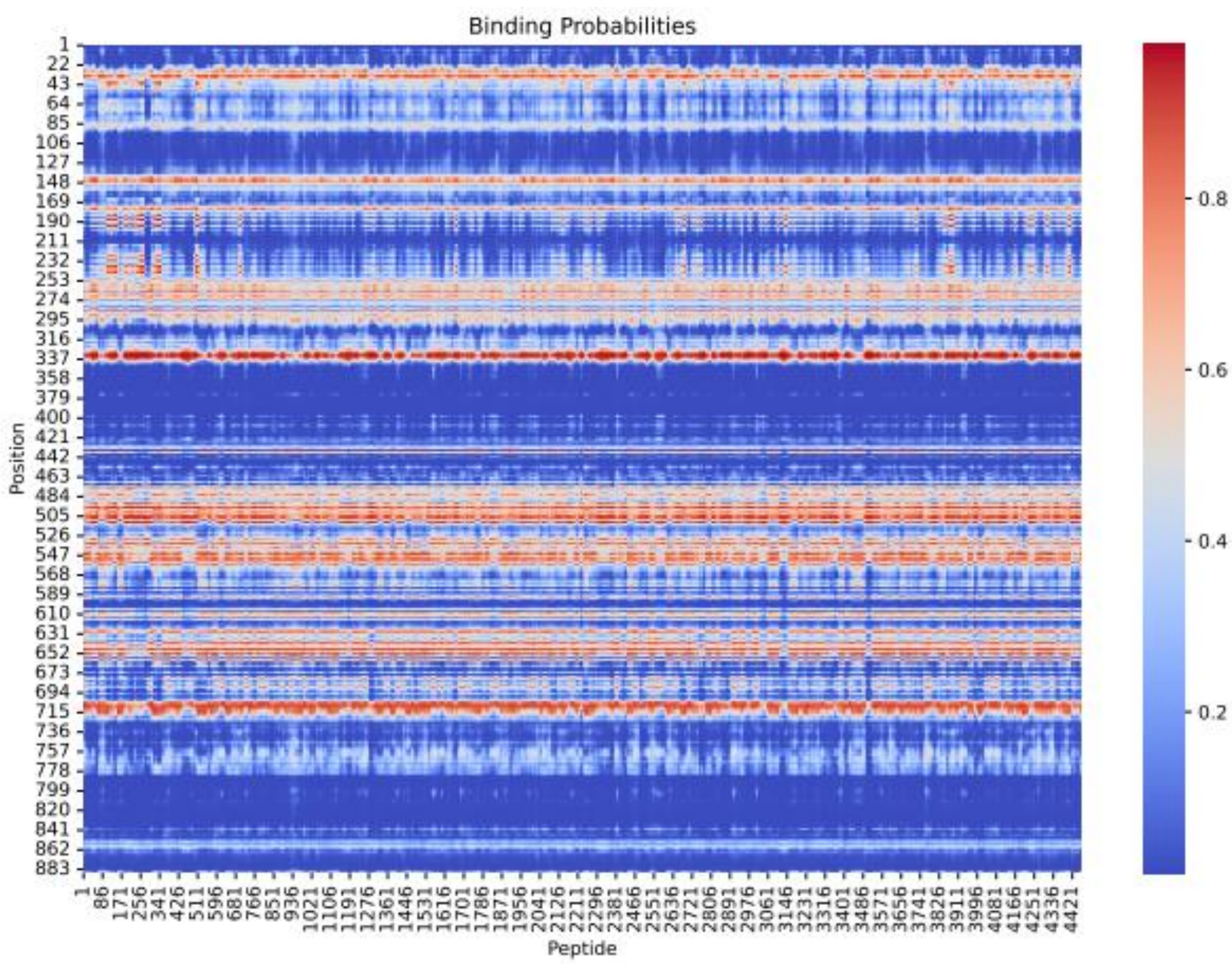

Supplementary Figure 4 | PepNN Predicted results PDE4A interaction with filamin A-derived peptides. The probabilities of filamin A-derived peptides (x axis) binding on PDE4A regions (y axis). The higher probabilities are shown in red.

Supplementary Table 2 This table compiles computationally predicted PDE4A interaction regions identified across AlphaFold3, peptide docking, and PepNN analyses. It summarizes the overlap between protein–protein interface predictions and peptide-binding pocket predictions used to prioritize candidate disruptor peptides.

PDE4A position	AlphaFold3 protein-protein interaction	AlphaFold3 protein-peptide interaction	PepNN: Peptide binding region		
			Amino acid	Probabilities	
				Mean	Peak
1-47	9-47	1-19	23-44	0.58	33 (1.00)
79-86	79-86		83-89	0.23	86 (0.51)
124-156	124-156	150-155	140-154	0.58	145 (1.00)
169-200	169-200	183-200	172-180	0.3	177 (1.00)
223-299	233-299	223-231	249-299	0.53	262 (1.00)
326-344	340-344				
348-354	348-354				
362-388	362-388				
397-412	397-412				
427-431		427-431	429-440	0.18	433 (0.78)
455-463	455-463	455-462			
473-580	526-535; 557-579	476-480; 484-492; 528-536; 574-580	473-564	0.56	495 (1.00)
603-662	603-615	603-627	606-619	0.27	613 (0.97)
	647-655		623-662	0.55	641 (1.00)
666-692	666-688		677-683	0.11	680 (0.57)
			686-692	0.27	689 (0.66)

700-721	704-718	704-716	700-721	0.65	706 (1.00)

Supplementary Table 2 | Computationally predicted interaction sites on PDE4A for filamin A and the disruptor peptide. AlphaFold3-predicted PDE4A-filamin A complex highlighting predicted binding regions on PDE4A. Residue pairs predicted to contribute to the interaction are listed. AlphaFold3-predicted PDE4A–peptide candidates complex highlighting predicted peptide–binding regions on PDE4A. PepNN predicted peptide binding pockets on PDE4A are listed. Mean is mean probability of 2628 peptides binding probabilities on PDE4A and peak is the highest probability of amino acid on PDE4A. Predicted interacting residues are listed.

References

1. Tibbo, A. J.; et al. Phosphodiesterase type 4 anchoring regulates cAMP signaling to Popeye domain-containing proteins. J. Mol. Cell. Cardiol. 2022, 165, 86–102.

2. Reichart, D.; et al. Pathogenic variants damage cell composition and single cell transcription in cardiomyopathies. Science 2022, 377, eabo1984.

3. Robinson, M. D.; McCarthy, D. J.; Smyth, G. K. edgeR: a Bioconductor package for differential expression analysis of digital gene expression data. Bioinformatics 2010, 26, 139–140.

4. Abramson, J.; et al. Accurate structure prediction of biomolecular interactions with AlphaFold 3. Nature 2024, 630, 493–500.

5. Zhang, Y.; Skolnick, J. TM-align: a protein structure alignment algorithm based on the TM-score. Nucleic Acids Res. 2005, 33, 2302–2309.

6. Bittrich, S.; et al. RCSB Protein Data Bank: exploring protein 3D similarities via comprehensive structural alignments. Bioinformatics 2024, 40.

7. Furmanova, K.; et al. COZOID: contact zone identifier for visual analysis of protein-protein interactions. BMC Bioinformatics 2018, 19, 125.

8. Cohen, M.; Potapov, V.; Schreiber, G. Four distances between pairs of amino acids provide a precise description of their interaction. PLoS Comput. Biol. 2009, 5, e1000470.

9. Tam, J. Z.; et al. Analysis of Protein-Protein Interactions for Intermolecular Bond Prediction. Molecules 2022, 27, 6178.

10. Yan, Y.; et al. The HDOCK server for integrated protein-protein docking. Nat. Protoc. 2020, 15, 1829–1852.

11. Yan, Y.; et al. HDOCK: a web server for protein-protein and protein-DNA/RNA docking based on a hybrid strategy. Nucleic Acids Res. 2017, 45, W365–W373.

12. Yan, Y.; et al. Addressing recent docking challenges: A hybrid strategy to integrate template-based and free protein-protein docking. Proteins 2017, 85, 497–512.

13. Huang, S. Y.; Zou, X. An iterative knowledge-based scoring function for protein-protein recognition. Proteins 2008, 72, 557–579.

14. Huang, S. Y.; Zou, X. A knowledge-based scoring function for protein-RNA interactions derived from a statistical mechanics-based iterative method. Nucleic Acids Res. 2014, 42, e55.

15. Abdin, O.; et al. PepNN: a deep attention model for the identification of peptide binding sites. Commun. Biol. 2022, 5, 503.

16. Sievers, F.; Higgins, D. G. Clustal Omega for making accurate alignments of many protein sequences. Protein Sci. 2018, 27, 135–145.

17. Zimmermann, L.; et al. A Completely Reimplemented MPI Bioinformatics Toolkit with a New HHpred Server at its Core. J. Mol. Biol. 2018, 430, 2237–2243.

18. Wright, P. T.; et al. Approaches to High-Throughput Analysis of Cardiomyocyte Contractility. Front. Physiol. 2020, 11, 612.

19. Sanchez-Alonso, J. L.; et al. Functional LTCC-beta(2)AR Complex Needs Caveolin-3 and Is Disrupted in Heart Failure. Circ. Res. 2023, 133, 120–137.

20. Sato, M.; et al. Enhancement of adenoviral gene transfer to adult rat cardiomyocytes in vivo by immobilization and ultrasound treatment of the heart. Gene Ther. 2005, 12, 936–941.

21. Wright, P. T.; et al. Cardiomyocyte Membrane Structure and cAMP Compartmentation Produce Anatomical Variation in beta(2)AR-cAMP Responsiveness in Murine Hearts. Cell Rep. 2018, 23, 459–469.

22. Bolte, S.; Cordelieres, F. P. A guided tour into subcellular colocalization analysis in light microscopy. J. Microsc. 2006, 224, 213–232.

23. Perera, R. K.; et al. Microdomain switch of cGMP-regulated phosphodiesterases leads to ANP-induced augmentation of beta-adrenoceptor-stimulated contractility in early cardiac hypertrophy. Circ. Res. 2015, 116, 1304–1311.

24. Fu, J.; et al. Stretch regulation of beta2-Adrenoceptor signalling in cardiomyocytes requires caveolae. Cardiovasc. Res. 2025, 121, 440–453.

25. Ponsioen, B.; et al. Detecting cAMP-induced Epac activation by fluorescence resonance energy transfer: Epac as a novel cAMP indicator. EMBO Rep. 2004, 5, 1176–1180.

26. Ramuz, M.; et al. A Software Tool for High-Throughput Real-Time Measurement of Intensity-Based Ratio-Metric FRET. Cells 2019, 8, 1541.
